# Effect of canagliflozin on left ventricular diastolic function in patients with type 2 diabetes

**DOI:** 10.1186/s12933-018-0717-9

**Published:** 2018-05-22

**Authors:** Daisuke Matsutani, Masaya Sakamoto, Yosuke Kayama, Norihiko Takeda, Ryuzo Horiuchi, Kazunori Utsunomiya

**Affiliations:** 1 Division of Diabetes, Metabolism and Endocrinology, Department of Internal Medicine, Jikei University School of Medicine, 3-25-8, Nishi-Shinbashi, Minato-ku, Tokyo, 105-8461 Japan; 2 Department of Cardiology, Jikei University School of Medicine, 3-25-8, Nishi-Shinbashi, Minato-ku, Tokyo, 105-8461 Japan; 3 Department of Cardiovascular Medicine, Graduate School of Medicine, The University of Tokyo, 7-3-1, Hongo, Bunkyo-ku, Tokyo, 113-8654 Japan; 4 Department of Pathology, Tsuruoka Kyoritsu Hospital, 9-34, Fumizonomachi, Tsuruoka-shi, Yamagata 997-0816 Japan

**Keywords:** Sodium-glucose cotransporter 2 inhibitors, Left ventricular diastolic function, Heart failure with preserved ejection fraction, Autonomic function, Type 2 diabetes mellitus

## Abstract

**Background:**

Type 2 diabetes mellitus (T2DM) greatly increases the risks of cardiovascular disease and heart failure. In particular, left ventricular diastolic dysfunction that develops from the early stages of T2DM is an important factor in the onset and exacerbation of heart failure. The effect of sodium-glucose cotransporter 2 inhibitors on left ventricular diastolic function has not been elucidated. We have performed the first prospective study on the effects of canagliflozin on left ventricular diastolic function in T2DM.

**Methods:**

This study was performed to evaluate the effects of additional treatment with canagliflozin for 3 months on left ventricular diastolic function in patients with T2DM. A total of 38 patients with T2DM were consecutively recruited for this study. Left ventricular diastolic function was assessed by echocardiography. The primary study outcome was a change in the septal E/e′ as a parameter of left ventricular diastolic function.

**Results:**

A total of 37 patients (25 males and 12 females) were included in the analysis. Mean age of participants was 64.2 ± 8.1 years (mean ± SD), mean duration of diabetes was 13.5 ± 8.1 years, and mean HbA1c was 7.9 ± 0.7%. Of the participants, 86.5% had hypertension, 100% had dyslipidemia, and 32.4% had cardiovascular disease. Canagliflozin significantly improved left ventricular diastolic function (septal E/e′ ratio 13.7 ± 3.5–12.1 ± 2.8, *p* = 0.001). Furthermore, among the various parameters that changed through the administration of canagliflozin, only changes in hemoglobin significantly correlated with changes in the septal E/e′ ratio (*p* = 0.002). In multiple regression analysis, changes in hemoglobin were also revealed to be an independent predictive factor for changes in the septal E/e′ ratio.

**Conclusions:**

This study showed for the first time that canagliflozin could improve left ventricular diastolic function within 3 months in patients with T2DM. The benefit was especially apparent in patients with substantially improved hemoglobin values.

*Trial registration* UMIN Clinical Trials Registry UMIN000028141

**Electronic supplementary material:**

The online version of this article (10.1186/s12933-018-0717-9) contains supplementary material, which is available to authorized users.

## Background

Type 2 diabetes mellitus (T2DM) greatly increases the risks of cardiovascular disease (CVD) and heart failure [1–3]. Especially, left ventricular diastolic dysfunction, which develops early in T2DM, is an important factor in the onset and exacerbation of heart failure [4–6]. T2DM has been shown to cause left ventricular dysfunction independently of glycemic control, hypertension, and coronary artery disease [7, 8], and its pathogenic mechanism involves enhanced oxidative stress and chronic inflammation, which accompanies hyperglycemia [9, 10]. However, glycemic control in those using anti-diabetic drugs was not found to be effective in improving left ventricular diastolic function [11]. Small-scale studies showed that dipeptidyl peptidase (DPP)-4 inhibitors and thiazolidinediones (TZD) were effective in improving left ventricular diastolic function [12, 13], but larger clinical studies showed exacerbation of heart failure with the use of such agents [14, 15]. The combined effect of factors such as aging, plasma glucose values, hypertension, and obesity might be partially responsible for the lack of an effective therapy for left ventricular diastolic dysfunction [4, 5, 8].

Recently, empagliflozin and canagliflozin, which are sodium-glucose cotransporter 2 (SGLT2) inhibitors, were reported to reduce all-cause mortality, cardiovascular mortality, and hospitalization due to heart failure in T2DM [16–19]. However, it is not clear yet how SGLT2 inhibitors reduced hospitalizations due to heart failure, which was shown in the early period of the EMPA-REG and CANVAS trials [16, 18]. Furthermore, the effect of SGLT2 inhibitors on left ventricular diastolic function has not been elucidated.

We have performed the first prospective study of the effects of canagliflozin on left ventricular diastolic function in patients with T2DM.

## Methods

### Study participants

This prospective single-center pilot study was performed to evaluate the effects of additional treatment with canagliflozin on left ventricular function in T2DM. Between July 2017 and December 2017, 38 diabetic patients who had inadequately controlled T2DM were consecutively recruited from outpatients at Tsuruoka Kyoritsu Hospital, Yamagata, Japan. All participants were evaluated at baseline and at 3 months after beginning additional treatment with canagliflozin. During the 6 months before the start of this study and the 3 months after the start of this study, participants had not changed their use of antidiabetic drugs or any other drugs that could affect glucose metabolism such as renin–angiotensin–aldosterone system (RAAS) inhibitors (angiotensin-converting enzyme inhibitors and/or angiotensin receptor blockers), beta blockers, diuretics, and statins. The inclusion criteria were as follows: (1) T2DM with HbA1c (NGSP) of ≥ 7.0%, < 10.5% and (2) experiencing no changes in antidiabetic drugs or any other drugs during the 6 months prior to the start of this study. Inclusion criteria also included (3) satisfaction of either (a) or (b) as follows: (a) age ≥ 45 and < 75 years without a history of CVD and with ≥ 2 of the following risk factors determined at the screening visit: duration of T2DM ≥ 10 years, systolic blood pressure (SBP) ≥ 140 mmHg (average of 3 readings at screening visit), taking at least one anti-hypertensive agent, current daily cigarette smoker (Brinkmann index ≥ 200), microalbuminuria or macroalbuminuria (microalbuminuria: urinary albumin excretion from 30 to 300 mg/g Cr; macroalbuminuria: urinary albumin excretion of ≥ 300 mg/g Cr); dyslipidemia (abnormal values for ≥ 1 among high-density lipoprotein [HDL] cholesterol < 40 mg/dL, low-density lipoprotein (LDL) cholesterol ≥ 120 mg/dL, and/or triglycerides (TG) ≥ 150 mg/dL); carotid intima media thickness ≥ 1.1 mm, and plaque positive; or (b) age ≥ 35 and < 75 years with a history of ≥ 1 of the following indicating CVD: old cerebral infarction, old myocardial infarction, hospital admission for unstable angina, chronic heart failure, coronary artery bypass graft, percutaneous coronary intervention (with or without stenting), peripheral revascularization (angioplasty or surgery), symptomatic with documented hemodynamically significant carotid or peripheral vascular disease, or amputation secondary to vascular disease. Patients were excluded based on: (1) age < 35 years or ≥ 75 years; (2) taking antipsychotics; (3) arrhythmia; (4) severe renal dysfunction (serum Cr ≥ 2.5 mg/dL); (5) severe liver dysfunction (3× upper limit of normal); (6) diabetic ketosis or diabetic coma; (7) insulin-dependent diabetes mellitus; (8) malignancy, or (9) acute heart failure or acute myocardial infarction. All participants underwent blood tests, including those for fasting plasma glucose, HbA1c, estimated glomerular filtration rate (eGFR), HDL cholesterol, LDL cholesterol, TG, brain natriuretic peptide (BNP), hemoglobin (Hb), and hematocrit (Hct). Clinical data (duration of diabetes; body mass index [BMI]; SBP; diastolic blood pressure [DBP]; heart rate [HR]; history of CVD; insulin therapy; and use of oral anti-diabetic drugs, anti-hypertensive agents, and lipid-lowering agents) were obtained from medical records and a questionnaire.

Thirty-seven patients were included in the analysis after excluding 1 with arrhythmias.

The study protocol was approved by the ethics committee of Tsuruoka Kyoritsu Hospital (approval number: 2017-01; date: 7th June, 2017), and the study was conducted according to the principles of the Helsinki Declaration II. This study was registered at the UMIN Clinical Trial Registry (UMIN000028141). All patients were informed of the purpose of the study after which consent was obtained.

### Analysis of echocardiographic findings

Standardized transthoracic and Doppler echocardiographic examinations were performed in all participants using commercially available equipment (iE33, Philips, Amsterdam, Netherlands). Left atrial diameter and end-diastolic and end-systolic left ventricular internal diameters were measured on a 2-dimensional guided M-mode recording. Left ventricular ejection fraction (EF) was assessed by the modified Simpson’s Biplane Method. Using two-dimensional echocardiograms the left ventricular mass index was determined by the area-length method [20]. To assess diastolic function, the following mitral pulse wave Doppler and tissue Doppler parameters were measured: peak early (E) and late (A) diastolic filling velocities, E/A ratio, deceleration time of E wave (DT), septal early diastolic mitral annular tissue velocity (septal e′), and lateral early diastolic mitral annular tissue velocity (lateral e′). We also calculated the E/e′ ratio by dividing the transmitral E peak by e′ [21].

### Assessment of baroreflex sensitivity (BRS)

Using the spontaneous sequence method, the beat-to-beat BP was measured for 15 min after 15 min of supine rest as the slope of the relationship between spontaneous changes in SBP and the pulse interval. Measurement was made using the second and third fingers of the right hand by the vascular unloading technique (Task Force Monitor, CNSystems, Graz, Austria). For calculation of BRS, the relative changes in SBP (mmHg) and the R–R interval (msec), which is expressed as the distance between corresponding QRS complexes, were considered according to the sequence method with cut-off points of 1 mmHg and 3 ms, respectively [22].

### Assessment of heart rate variability (HRV)

A 3-lead ECG with a sampling frequency of 1000 Hz was recorded for 15 min after the participant had rested for 15 min. The HRV was calculated during the test and included all recorded R–R intervals. HRV was calculated by the frequency domain method using a power spectral analysis of rhythmic oscillations of the R–R interval. The relative contributions of the low frequency (LF) power (0.04–0.15 Hz), high frequency (HF) power (0.15–0.4 Hz), and very-low-frequency (VLF) power (0–0.04 Hz) to total power were calculated as follows: *LF normalized units (LFnu%) *=* LF absolute power* (ms^2^)*/total power* (ms^2^)* − VLF power* (ms^2^)×100 *and HFnu% *=* HF absolute power* (ms^2^)*/total power* (ms^2^)* − VLF* (ms^2^)×100. The LFnu%/HFnu% ratio was considered to indicate sympathetic/vagal tone. All calculations were performed using the Task Force Monitor (CNSystems, Graz, Austria) [23].

### Study outcome

The primary study outcome was a change in the septal E/e` as a parameter of left ventricular diastolic function. Secondary endpoints included changes in the following variables at the end of the 12-week treatment relative to the baseline: (1) echocardiographic parameters: left atrial diameter, left ventricular end-diastolic diameter, left ventricular end-systolic diameter, EF, left ventricular mass index, E, A, E/A, DcT, septal e′, lateral e′, and lateral E/e′; (2) autonomic function: BRS and LF/HF; (3) glycemic control variables: fasting plasma glucose and HbA1c level; (4) lipid metabolism variables: TG, LDL cholesterol, and HDL cholesterol; (5) BMI; (6) SBP and DBP, (7) HR; (8) BNP; (9) Hb and Hct; and (10) eGFR.

### Sample size and statistical analyses

Because there has been no report on the effect of SGLT-2 inhibitors to improve the septal E/e′, we used information from a previous report on the effect of another drug on the septal E/e′ (approximately a 1.5 reduction) to estimate the required sample size [12]. Sample size estimates were based on the following: standard deviation, 3; α-level, 0.05; and power, 80%. Sample size was estimated to be 33 people. Assuming a dropout rate of 10%, the target number of patients was therefore set at 38 patients.

Data analyses were performed using the Statistical Package for the Social Sciences 22.0 software (IBM, Armonk, NY, USA). Patients’ characteristics and results are presented as mean ± SD, mean ± standard error, or median with interquartile range (IQR) as appropriate according to the data distribution. Comparison of variables between baseline and 3 months after treatment were made using the paired *t* test or Wilcoxon signed-rank test (Table 2). Pearson’s correlation analysis or Spearman’s rank correlation coefficient test was used for single correlations (Table 3). Multiple-linear regression was used to assess individual and cumulative effects of ΔHb, age, sex, SBP, eGFR, and HR on the Δseptal E/e′ ratio. Independent variables were selected among variables that were significantly correlated with the Δseptal E/e′ ratio (Table 4). The delta (∆) values for BRS and LF/HF were calculated according to values at 3 months after treatment-value at baseline. All other delta values were calculated as [(value at 3 months after treatment-value at baseline) × 100]/value at baseline (%). As shown in Fig. 2 and Table 5, the baseline septal E/e′ ratio was divided into tertiles (T1 < 12, T2 ≥ 12- < 16, T3 ≥ 16). The Jonckheere trend test was used to test for linear trends in Δseptal E/e′ ratio in relation to baseline septal E/e′ ratio tertiles. The analysis of variance (ANOVA) was used to compare Δseptal E/e′ ratios among participants with various baseline septal E/e′ ratio tertiles. In ANOVA, the Tukey post hoc test was also used to compare Δseptal E/e′ ratios among different baseline septal E/e′ ratio groups. As shown in Table 6, the effects of canagliflozin on Δseptal E/e′ ratios were analyzed for the following subgroups: sex, history of hypertension, presence of CVD, insulin use, sulfonylurea use, metformin use, DPP-4 inhibitor use, calcium channel blocker use, RAAS inhibitor use, beta blocker use, and diuretic use. In the subgroup analysis, mean Δseptal E/e′ was compared using Student’s *t* test. Hypertension was defined as follows: SBP ≥ 140 mmHg, DBP ≥ 90 mmHg and/or the use of at least one anti-hypertensive agent. CVD was considered to be present if any of the following had occurred: cerebral infarction, myocardial infarction, hospital admission for unstable angina, heart failure, coronary artery bypass graft, percutaneous coronary intervention (with or without stenting), peripheral revascularization (angioplasty or surgery), symptoms consistent with documented hemodynamically significant carotid or peripheral vascular disease, and/or amputation secondary to vascular disease. As shown in Additional file 1: Table S1, participants were divided into a primary prevention group without a history of CVD, a secondary prevention group with a history of CVD, and an overall population group. In each group, comparison of variables between baseline and 3 months after treatment were made using the paired *t* test. The Student’s *t* test was used to compare mean Δseptal E/e′ in the primary prevention group and secondary prevention group. A *p* value < 0.05 was considered significant.

## Results

### Baseline characteristics of study participants

A total of 37 patients (25 males and 12 females) were included in the analysis. Table 1 shows the baseline clinical, anthropometric, and pharmacologic data on the study participants. Mean age of participants was 64.2 ± 8.1 years (mean ± SD), mean duration of diabetes was 13.5 ± 8.1 years, and mean HbA1c was 7.9 ± 0.7%. Of the participants, 86.5% had hypertension, 100% had dyslipidemia, and 32.4% had CVD. At baseline, 32.4% of patients were on insulin, 37.8% on sulfonylureas, 54.1% on metformin, 43.2% on DPP-4 inhibitors, 45.9% on calcium-channel blockers, 45.9% on RAAS inhibitors (angiotensin-converting enzyme inhibitors and/or angiotensin receptor blockers), 13.5% on beta blockers, 10.8% on diuretics, 48.6% on statins, and fibrates on 2.7%.

**Table 1:** Baseline characteristics of the study population

Age, (years)	64.2 ± 8.1
No. patients	37
Sex, male/female	25/12
Duration of diabetes, (years)	13.5 ± 8.1
BMI, (kg/m^2^)	27.1 ± 4.6
Fasting plasma glucose, (mg/dL)	146.3 ± 28.8
HbA1c, (%)	7.9 ± 0.7
Hypertension, n (%)	32 (86.5)
Dyslipidemia, n (%)	37 (100)
History of cardiovascular disease, n (%)	12 (32.4)
Oral anti-diabetic drugs, n (%)	34 (91.9)
Insulin, n (%)	12 (32.4)
Sulfonylureas, n (%)	14 (37.8)
Glinides, n (%)	1 (2.7)
Metformin, n (%)	20 (54.1)
Thiazolidinediones, n (%)	5 (13.5)
DPP-4 inhibitors, n (%)	16 (43.2)
GLP-1 receptor agonists, n (%)	2 (5.4)
α-glucosidase inhibitors, n (%)	10 (27)
Anti-hypertensive agents, n (%)	25 (67.6)
Calcium channel blockers, n (%)	17 (45.9)
RAAS inhibitors, n (%)	17 (45.9)
Beta blockers, n (%)	5 (13.5)
Diuretics, n (%)	4 (10.8)
Lipid-lowering agents, n (%)	18 (48.6)
Statins, n (%)	18 (48.6)
Fibrates, n (%)	1 (2.7)

Values are mean ± SD or no. (%)

*BMI* body mass index; *DPP* dipeptidyl peptidase-4; *GLP* glucagon-like peptide; *RAAS* renin–angiotensin–aldosterone system

### Comparison of values from baseline to after 3 months

**Fig. 1:**
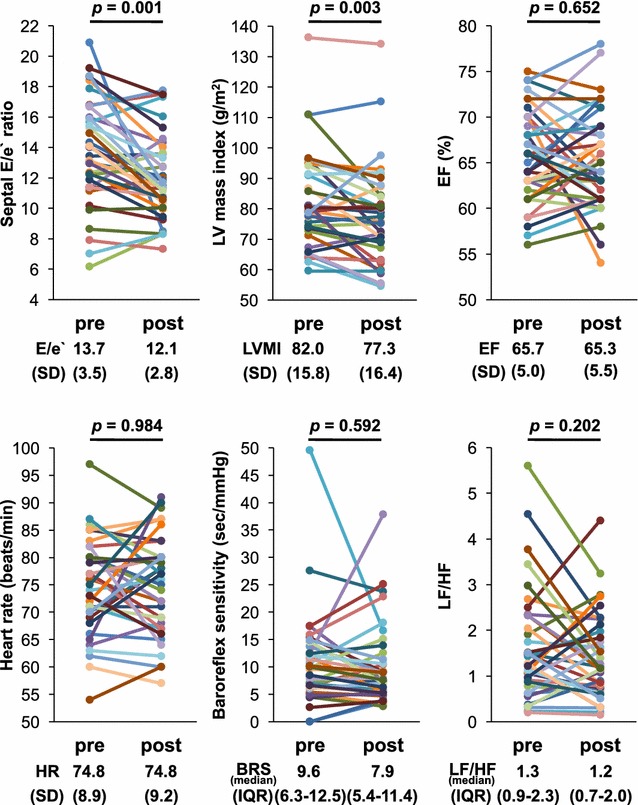
Figure title: Septal E/e′ ratio, left ventricular mass index, EF, heart rate, baroreflex sensitivity, and LF/HF at baseline (pre-canagliflozin) and at the 3-month follow-up (post-canagliflozin). Data are mean ± SD or median (25th–75th percentiles). *E* velocity of early mitral flow; *e′*, early peak velocity of septal annulus; *LVMI* left ventricular mass index; *EF* ejection fraction; *BRS* baroreflex sensitivity; *LF/HF* low frequency/high frequency; *SD* standard deviation; *IQR* interquartile range

Compared to baseline levels, decreases were found at 3 months in mean fasting plasma glucose (146.3 ± 28.8 mg/dL to 125.9 ± 24.6 mg/dL, *p* = 0.001), mean HbA1c (7.9 ± 0.7% to 7.1 ± 0.6%, *p* < 0.001), mean SBP (134.1 ± 14.3 mmHg to 130.3 ± 15.1 mmHg, *p* = 0.028), mean left ventricular mass index (82.0 ± 15.8 g/m^2^ to 77.3 ± 16.4 g/m^2^, *p *= 0.003), mean septal E/e′ ratio (13.7 ± 3.5 to 12.1 ± 2.8, *p* = 0.001), and mean lateral E/e′ ratio (9.7 ± 2.9–8.3 ± 1.9, *p* < 0.001). Increases after 3 months of treatment were identified in mean Hb (13.9 ± 1.2 g/dL to 14.7 ± 1.4 g/dL, *p* < 0.001) and mean Hct (40.7 ± 3.3% to 44.0 ± 3.7%, *p* < 0.001). There were no differences from baseline values to the end of the 3-month treatment period in median BNP (11.4 [IQR 9.1–20.6] pg/mL to 10.6 [IQR 6.3–15.8] pg/dL, *p* = 0.061), mean EF (65.7 ± 5.0% to 65.3 ± 5.5%, *p* = 0.652), median BRS (9.6 [IQR 6.3-12.5] ms/mmHg to 7.9 [IQR 5.4–11.4] ms/mmHg, *p* = 0.592), and median LF/HF (1.3 [IQR 0.9–2.3] to 1.2 [IQR 0.7–2.0], *p *= 0.202) (Fig. 1, Table 2).

**Table 2:** Comparison of variables between baseline and 3 months after treatment

Variables	Pre canagliflozin	Post canagliflozin	*p* value
Fasting blood glucose (mg/dL)	146.3 ± 28.8	125.9 ± 24.6	0.001
HbA1c (%)	7.9 ± 0.7	7.1 ± 0.6	< 0.001
BMI (kg/m^2^)	27.1 ± 4.6	26.5 ± 4.3	< 0.001
Blood pressure (mmHg)			
Systolic	134.1 ± 14.3	130.3 ± 15.1	0.028
Diastolic	80.8 ± 9.9	81.4 ± 9.5	0.627
Heart rate (beats/min)	74.8 ± 8.9	74.8 ± 9.2	0.984
Lipid profile (mg/dL)			
Triglycerides	146 (101–190)	114 (85–148)	< 0.001
LDL cholesterol	111.2 ± 32.7	113.9 ± 31.0	0.516
HDL cholesterol	47.9 ± 10.5	53.9 ± 11.9	< 0.001
eGFR (mL/min/1.73 m^2^)	76.1 ± 19.5	71.4 ± 16.9	0.001
BNP (pg/mL)	11.4 (9.1–20.6)	10.6 (6.3–15.8)	0.061
Hemoglobin (g/dL)	13.9 ± 1.2	14.7 ± 1.4	< 0.001
Hematocrit (%)	40.7 ± 3.3	44.0 ± 3.7	< 0.001
Echocardiographics			
Left atrial diameter (mm)	36.4 ± 5.3	36.5 ± 5.2	0.789
LV end-diastolic diameter (mm)	47.8 ± 5.4	47.2 ± 4.8	0.253
LV end-systolic diameter (mm)	31.2 ± 5.1	30.5 ± 4.0	0.226
EF (%)	65.7 ± 5.0	65.3 ± 5.5	0.652
LV mass index (g/m^2^)	82.0 ± 15.8	77.3 ± 16.4	0.003
E (m/s)	0.7 ± 0.1	0.6 ± 0.1	0.013
A (m/s)	0.9 ± 0.2	0.2 ± 0.2	0.053
E/A	0.8 ± 0.2	0.8 ± 0.2	0.446
DcT (ms)	265.5 ± 53.4	276.2 ± 58.0	0.298
Septal e′ (cm/s)	5.3 ± 1.2	5.4 ± 1.2	0.294
Lateral e′ (cm/s)	7.5 ± 1.6	7.8 ± 1.6	0.097
Septal E/e′ ratio	13.7 ± 3.5	12.1 ± 2.8	0.001
Lateral E/e′ ratio	9.7 ± 2.9	8.3 ± 1.9	< 0.001
Autonomic function			
BRS (s/mmHg)	9.6 (6.3–12.5)	7.9 (5.4–11.4)	0.592
LF/HF	1.3 (0.9–2.3)	1.2 (0.7–2.0)	0.202

Data are mean ± SD or median (25th–75th percentiles)

*BMI* body mass index; *LDL* low density lipoprotein; *HDL* high density lipoprotein; *eGFR* estimated glomerular filtration rate; *BNP* brain natriuretic peptide; *LV* left ventricular; *EF* ejection fraction; *E* velocity of early mitral flow; *A* velocity of late mitral flow; *DcT* deceleration time of early mitral inflow; *e′* early peak velocity of annulus; *BRS* baroreflex sensitivity; *LF/HF* low frequency/high frequency

### Univariate correlates of changes in the septal E/e′ ratio

Correlation analysis showed that the Δseptal E/e′ ratio was correlated with the baseline septal E/e′ ratio (*r* = − 0.618, *p* = 0.000), age (*r* = − 0.464, *p* = 0.004), baseline SBP (*r* = − 0.335, *p* = 0.042), baseline HR (*r* = 0.359, *p* = 0.029), baseline eGFR (*r* = 0.355, *p* = 0.031), and ΔHb (*r* = − 0.496, *p* = 0.002) (Table 3).

**Table 3:** Univariate correlates of change in septal E/e′ ratio vs. baseline and change in variables

Variables	Δseptal E/e′ vs. baseline variables		Δseptal E/e′ vs. Δvariables	
	*r*	*p*	*r*	*p*
Age (years)	− 0.464	0.004	–	–
Duration of diabetes (years)	0.021	0.902	–	–
Septal E/e′ ratio	− 0.618	< 0.001	–	–
LV mass index (g/m^2^)	− 0.252	0.133	− 0.158	0.351
BMI (kg/m^2^)	− 0.054	0.753	0.034	0.840
Fasting plasma glucose (mg/dL)	− 0.009	0.960	− 0.277	0.097
HbA1c (%)	− 0.009	0.957	− 0.060	0.725
SBP (mmHg)	− 0.335	0.042	0.066	0.699
DBP (mmHg)	− 0.022	0.899	− 0.092	0.588
Heart rate (beats/min)	0.359	0.029	− 0.223	0.184
BNP (pg/mL)	0.074	0.665	− 0.084	0.623
Hemoglobin (g/dL)	0.092	0.588	− 0.496	0.002
Hematocrit (%)	0.095	0.575	− 0.265	0.112
eGFR (mL/min/1.73 m^2^)	0.355	0.031	0.016	0.924
Triglycerides (mg/dL)	− 0.115	0.497	− 0.159	0.346
LDL cholesterol (mg/dL)	0.036	0.833	− 0.084	0.621
HDL cholesterol (mg/dL)	0.124	0.463	0.039	0.819
BRS (s/mmHg)	0.232	0.167	0.266	0.111
LF/HF	0.211	0.211	− 0.322	0.052

The delta (Δ) values for baroreflex sensitivity, and LF/HF were calculated as values at 3 months after treatment-value at baseline. All other delta values were calculated as [(value at 3 months after treatment − value at baseline) × 100]/value at baseline (%)

*E* velocity of early mitral flow; *e′* early peak velocity of annulus; *LV* left ventricular; *BMI* body mass index; *SBP* systolic blood pressure; *DBP* diastolic blood pressure; *BNP* brain natriuretic peptide; *eGFR* estimated glomerular filtration rate; *LDL* low density lipoprotein; *HDL* high density lipoprotein; *BRS* baroreflex sensitivity; *LF/HF* low frequency/high frequency

### Multivariate analysis of changes in the septal E/e′ ratio

Multiple regression analysis showed that ΔHb were inversely related to the Δseptal E/e′ ratio. These findings remained after adjusting the Δseptal E/e′ ratio for age, sex, SBP, eGFR, and HR (Table 4).

**Table 4:** Multiple regression analysis of changes in the septal E/e′ ratio

Independent variables	Model 1		Model 2		Model 3	
	*β*	*p*	*β*	*p*	*β*	*p*
ΔHemoglobin (g/dL)	− 0.357	0.024	− 0.374	0.017	− 0.424	0.005
Age (years)	− 0.266	0.086	− 0.192	0.238	− 0.174	0.244
Sex (male/female)	− 0.195	0.186	− 0.265	0.063	− 0.266	0.049
SBP (mmHg)	− 0.186	0.206				
eGFR (mL/min/1.73 m^2^)			0.188	0.213		
Heart rate (beats/min)					0.311	0.024

Dependent variable was Δ septal E/e′, and the independent variables were Model 1, Model 2, and Model 3. Model 1: Δhemoglobin, age, sex, and SBP; Model 2: Δhemoglobin, age, sex, and eGFR; Model 3: Δhemoglobin, age, sex, and heart rate. Model 1: R-squared 0.420, adjusted R-squared 0.348; Model 2: R-squared 0.419, adjusted R-squared 0.347; Model 3: R-squared 0.481, adjusted R-squared 0.416

*E* velocity of early mitral flow; *e′* early peak velocity of septal annulus; *SBP* systolic blood pressure; *eGFR* estimated glomerular filtration rate

### Comparison of changes in the septal E/e′ ratio with the baseline septal E/e′ ratio

**Fig. 2:**
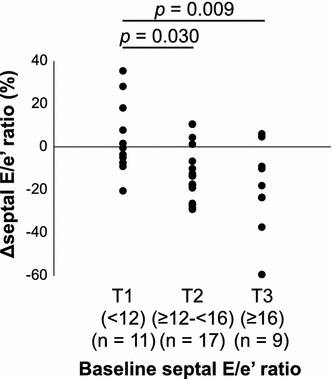
Comparison of changes in septal E/e′ ratio with baseline septal E/e′ ratio based on ANOVA. Changes in the septal E/e′ ratio divided according to baseline septal E/e′ ratio tertiles. Tukey post hoc test compared with T1. *E* velocity of early mitral flow; *e′* early peak velocity of septal annulus

Figure 2 and Table 5 show the comparisons of Δseptal E/e′ ratio among participants with various baseline septal E/e′ ratios according to tertiles based on ANOVA. There was a significant difference in Δseptal E/e′ ratios among these three groups. The results were then analyzed by the Tukey post hoc test. The T2 (*p* = 0.03) and T3 (*p* = 0.009) groups had decreased septal E/e′ ratios in comparison with the T1 group. This observation was confirmed by the Jonckheere trend test: Δseptal E/e′ ratio (*p* = 0.008) was significantly correlated with tertiles of baseline septal E/e′ ratios.

**Table 5:** Comparison of Δseptal E/e′ ratios with baseline septal E/e′ ratios based on ANOVA

Baseline septal E/e′ ratio tertiles		T1 (< 12) (n = 11)	T2 (≥ 12– < 16) (n = 17)	T3 (≥ 16) (n = 9)	ANOVA *p* value	Test for trend *p* value
Δseptal E/e′ ratio (%)		4.1 ± 0.3	− 12.6 ± 0.4	− 19.0 ± 0.4	0.007	0.008
*p* value			0.030	0.009		
Age (years)		58.6 ± 7.7	64.4 ± 7.5	70.6 ± 4.2		
Duration of diabetes (years)		10.5 ± 5.7	13.1 ± 8.6	17.9 ± 8.7		
SBP (mmHg)		127.9 ± 10.7	132.3 ± 13.4	145.1 ± 14.8		
Heart rate (beats/min)		78.2 ± 7.9	72.1 ± 9.2	76.0 ± 8.6		
eGFR (mL/min/1.73 m^2^)		84.6 ± 23.3	76.3 ± 18.9	65.1 ± 9.4		
Δhemoglobin (%)		5.0 ± 3.5	6.1 ± 5.1	6.4 ± 4.6		

Δseptal E/e′ ratios are mean ± SE. All other values are mean ± SD. Changes in the septal E/e′ ratio were divided according to baseline septal E/e′ ratio tertiles. Results of the Tukey post hoc test compared with T1

*E* velocity of early mitral flow; *e′* early peak velocity of septal annulus; *SBP* systolic blood pressure; *eGFR* estimated glomerular filtration rate

### Effects of canagliflozin on septal E/e′ ratio in subgroups of the study population

The septal E/e′ ratio was significantly improved in patients using sulfonylurea (*p* = 0.006) or a DPP-4 inhibitor (*p* = 0.014) at baseline. However, there was no significant difference in patients grouped according to sex, history of hypertension, presence of CVD, insulin use, metformin use, calcium channel blocker use, RAAS inhibitor use, beta blocker use, and diuretic use (Table 6).

**Table 6:** Effects of canagliflozin on the septal E/e′ ratio in subgroups of study patients

Subgroup	No. (%)	Δseptal E/e′ ratio (%)	*p* value for interaction
Sex			
Male	25 (68)	− 5.6 ± 3.1	0.076
Female	12 (32)	− 16.9 ± 6.2	
Hypertension			
Yes	32 (86)	− 9.4 ± 3.3	0.883
No	5 (14)	− 8.1 ± 7.5	
History of cardiovascular disease			
Yes	12 (32)	− 14.0 ± 3.4	0.277
No	25 (68)	− 7.0 ± 3.4	
Insulin use			
Yes	12 (32)	− 2.8 ± 5.7	0.141
No	25 (68)	− 12.3 ± 3.4	
Sulfonylurea use			
Yes	14 (38)	− 19.4 ± 4.7	0.006
No	23 (62)	− 3.1 ± 3.3	
Metformin use			
Yes	20 (54)	− 10.4 ± 4.4	0.688
No	17 (46)	− 7.9 ± 4.0	
DPP-4 inhibitor use			
Yes	16 (43)	− 17.5 ± 4.3	0.014
No	21 (57)	− 3.0 ± 3.6	
Calcium channel blocker use			
Yes	17 (46)	− 13.2 ± 4.4	0.230
No	20 (54)	− 5.9 ± 4.0	
RAAS inhibitor use			
Yes	17 (46)	− 13.8 ± 3.3	0.157
No	20 (54)	− 5.3 ± 4.7	
Beta blocker use			
Yes	5 (14)	− 21.9 ± 4.5	0.092
No	32 (86)	− 7.2 ± 3.3	
Diuretic use			
Yes	4 (11)	− 25.7 ± 4.4	0.053
No	33 (89)	− 7.2 ± 3.1	

Values are mean ± SE or no. (%)

*DPP* dipeptidyl peptidase-4; *RAAS* renin–angiotensin–aldosterone system

## Discussion

This is the first prospective study of the effects of canagliflozin on left ventricular diastolic function in patients with T2DM who were at high risk of a cardiovascular event. Furthermore, among various parameters that changed through the administration of canagliflozin, only ΔHb significantly correlated with the Δseptal E/e′ ratio. Multiple regression analysis also revealed that ΔHb was an independent predictive factor for the Δseptal E/e′ ratio. On the other hand, canagliflozin caused no exacerbation of autonomic function as assessed by BRS and frequency domain analysis of HRV.

SGLT2 inhibitors were reported to increase Hb, and the involvement of erythropoietin in the mechanism for this increase was reported [24–26]. Inhibited reabsorption of glucose from the proximal tubule was suggested to reduce oxygen consumption in proximal tubule cells, accelerate the recovery of erythropoietin production from interstitial fibroblasts, and thus cause increases in Hb [25, 27]. Reports showed that erythropoietin had cardioprotective effects [28–31] and that left ventricular diastolic function was improved in patients whose Hb was increased by an erythropoiesis-stimulating agent [32, 33]. In addition, increases in Hb improve the oxygen supply to tissues, which also results in improved left ventricular diastolic function [34, 35]. Another important mechanism for the increased Hb values is a reduction in plasma volume due to osmotic diuresis and the natriuretic effect [26, 36]. Chronic expanded plasma volume is known to increase the burden on the myocardium and trigger cardiac disorders. In patients having heart or renal failure, including those with concomitant diabetes, increases in Hb through decreased plasma volume were reported to correlate with improved mortality [37, 38]. As SGLT2 inhibitors have been considered to reduce the cardiac workload by decreasing plasma volume [39, 40], in the present study, decreased plasma volume may have reduced the preload and afterload, resulting in improved left ventricular diastolic function. Such improvement might also be attributed to the simultaneous effects of decreased BP [41], reduced body weight, less visceral fat [42, 43], and improvements in endothelial dysfunction [44, 45], systemic micro-inflammation [43], cardiac injury [46], left ventricular hypertrophy, etc. Further investigation is needed to elucidate the mechanism for the improved left ventricular diastolic function resulting from the administration of an SGLT2 inhibitor.

Reductions in BP and plasma volume by an SGLT2 inhibitor diminish the cardiac load, but these agents could possibly have the adverse effect of enhancing sympathetic nervous system activity. Increased sympathetic nervous system activity has important roles in the feedback mechanism of lowered BP and reduced plasma volume; but over the long term, it is known to cause increases in heart load due to an elevated HR and fluid retention and thus becomes a factor in left ventricular diastolic dysfunction. Loop diuretics used for heart failure patients are known to increase sympathetic nervous system activity by reducing plasma volume and thus to adversely affect the long-term outcome [47]. In this study, canagliflozin did not exacerbate the autonomic function assessed by BRS and frequency domain analysis of HRV. As an SGLT2 inhibitor was reported to improve BRS in an animal study [48], research on the effects of long-term administration of SGLT2 inhibitors on autonomic function are awaited.

In our subgroup analysis, the improvement in left ventricular diastolic function was significantly greater in those who took sulfonylurea before the start of the study and continued thereafter than in those not given this agent. As sulfonylurea was reported to be involved in left ventricular diastolic dysfunction [11] and has attenuated cardioprotective effects [49], it is possible that canagliflozin compensated for the dysfunction caused by the administration of sulfonylurea while reducing the load on the heart. Furthermore, our study patients who were provided with a DPP-4 inhibitor had a significantly greater improvement in their left ventricular diastolic function than those not provided with this drug. A DPP-4 inhibitor is an oral anti-hyperglycemic agent that has many advantages, such as an antiarteriosclerotic effect [50] and vascular endothelial function-improving effect [51], but increased hospitalizations due to heart failure have been noted [15]. We found that combination therapy of a DPP-4 inhibitor with the SGLT2 inhibitor improved left ventricular diastolic function possibly because the SGLT2 inhibitor compensated for some of the concerns regarding DPP-4 inhibitors. In this study, a significant correlation (*r* = − 0.464, *p* = 0.004) was observed between age and improvement in left ventricular diastolic function. Patients administered sulfonylurea (*p* = 0.025) or a DPP-4 inhibitor (*p* = 0.004) were significantly older than those with whom they were compared; thus the results possibly reflect these differences.

Recently, although empagliflozin was reported to have improved left ventricular diastolic function in patients with T2DM with a history of CVD [52], this is the first report showing the possibility of canagliflozin improving left ventricular diastolic function in patients with T2DM who were mainly grouped according to primary prevention. As the sub-analysis in the CANVAS trial showed a similar inhibitory effect on heart failure-associated hospitalizations between the primary and secondary prevention participants [19] and canagliflozin significantly suppressed such hospitalizations compared to a DPP-4 inhibitor and a glucagon-like peptide-1 receptor agonist [53], it is possible that improvement in left ventricular diastolic function was involved in those results. Also, a recent sub-analysis in the EMPA-REG trial indicated that Hb increases were involved in suppression of cardiovascular events independently of lowered plasma glucose and decreased BP, suggesting the involvement of improved left ventricular diastolic function via rises in Hb [54].

The incidence of heart failure, particularly heart failure with preserved ejection fraction (HFpEF) for which diastolic dysfunction is a major cause, is rapidly increasing globally with aging of the population [55, 56]. Thus establishment of therapy for such conditions is imperative. However, the outcome of HFpEF cannot be improved even with an angiotensin-converting-enzyme inhibitor, angiotensin receptor blocker, beta blocker, or aldosterone antagonist [57–60], all of which are effective for improving the outcome in heart failure with a reduced ejection fraction (HFrEF) [61]. A possible reason may be that HFpEF develops due to multiple factors, such as diabetes, hypertension, obesity, and renal dysfunction; optimal treatment of these diseases is likely important in improving left ventricular diastolic function and outcome in HFpEF patients. An SGLT2 inhibitor is likely to be a promising drug in the prevention of HFpEF and improving the outcome of HFpEF by improving left ventricular diastolic function in patients with T2DM. In addition, our results suggested that increases in Hb are involved in improved left ventricular diastolic function and can be used as a predictive marker of such improvement.

This study has three limitations. First, although this was a prospective study, the number of study patients was small and this was uncontrolled observational study. Therefore, larger randomized controlled trials are needed. Second, we could not verify whether the improvement in diastolic function was solely attributable to canagliflozin. Finally, the relationship between the administration of canagliflozin and cardiovascular events could not be verified due to the short research period.

## Conclusions

This study showed for the first time that canagliflozin could improve left ventricular diastolic function in patients with T2DM. The benefit was especially apparent in patients with substantial improvement in Hb values. This result suggests that canagliflozin may have a role in preventing cardiovascular events and heart failure.

## Additional file

**Additional file 1: Table S1.** Comparison of echocardiographic findings between baseline and at the end of the 3-months study period in the total population, the primary, and secondary prevention.

## Abbreviations

T2DM: type 2 diabetes mellitus
SGLT: sodium-glucose cotransporter
CVD: cardiovascular disease
BRS: baroreflex sensitivity
HFpEF: heart failure with preserved ejection fraction
HFrEF: heart failure with reduced ejection fraction

## References

[1] Chen G, McAlister FA, Walker RL, Hemmelgarn BR, Campbell NR (2011). Cardiovascular outcomes in Framingham participants with diabetes: the importance of blood pressure. Hypertension.

[2] Vazquez-Benitez G, Desai JR, Xu S, Goodrich GK, Schroeder EB, Nichols GA, Segal J, Butler MG, Karter AJ, Steiner JF (2015). Preventable major cardiovascular events associated with uncontrolled glucose, blood pressure, and lipids and active smoking in adults with diabetes with and without cardiovascular disease: a contemporary analysis. Diabetes Care.

[3] Kannel WB, McGee DL (1979). Diabetes and cardiovascular disease. The Framingham study. Jama.

[4] Klapholz M, Maurer M, Lowe AM, Messineo F, Meisner JS, Mitchell J, Kalman J, Phillips RA, Steingart R, Brown EJ (2004). Hospitalization for heart failure in the presence of a normal left ventricular ejection fraction: results of the New York Heart Failure Registry. J Am Coll Cardiol.

[5] Fischer M, Baessler A, Hense HW, Hengstenberg C, Muscholl M, Holmer S, Doring A, Broeckel U, Riegger G, Schunkert H (2003). Prevalence of left ventricular diastolic dysfunction in the community. Results from a Doppler echocardiographic-based survey of a population sample. Eur Heart J.

[6] Paulus WJ, Tschope C, Sanderson JE, Rusconi C, Flachskampf FA, Rademakers FE, Marino P, Smiseth OA, De Keulenaer G, Leite-Moreira AF (2007). How to diagnose diastolic heart failure: a consensus statement on the diagnosis of heart failure with normal left ventricular ejection fraction by the Heart Failure and Echocardiography Associations of the European Society of Cardiology. Eur Heart J.

[7] van den Hurk K, Alssema M, Kamp O, Henry RM, Stehouwer CD, Smulders YM, Nijpels G, Paulus WJ, Dekker JM (2012). Independent associations of glucose status and arterial stiffness with left ventricular diastolic dysfunction: an 8-year follow-up of the Hoorn Study. Diabetes Care.

[8] From AM, Scott CG, Chen HH (2009). Changes in diastolic dysfunction in diabetes mellitus over time. Am J Cardiol.

[9] Zhang X, Chen C (2012). A new insight of mechanisms, diagnosis and treatment of diabetic cardiomyopathy. Endocrine.

[10] Ernande L, Derumeaux G (2012). Diabetic cardiomyopathy: myth or reality?. Arch Cardiovasc Dis.

[11] Inoue T, Maeda Y, Sonoda N, Sasaki S, Kabemura T, Kobayashi K, Inoguchi T (2016). Hyperinsulinemia and sulfonylurea use are independently associated with left ventricular diastolic dysfunction in patients with type 2 diabetes mellitus with suboptimal blood glucose control. BMJ Open Diabetes Res Care.

[12] Hashikata T, Yamaoka-Tojo M, Kakizaki R, Nemoto T, Fujiyoshi K, Namba S, Kitasato L, Hashimoto T, Kameda R, Maekawa E (2016). Teneligliptin improves left ventricular diastolic function and endothelial function in patients with diabetes. Heart Vessels.

[13] Horio T, Suzuki M, Suzuki K, Takamisawa I, Hiuge A, Kamide K, Takiuchi S, Iwashima Y, Kihara S, Funahashi T (2005). Pioglitazone improves left ventricular diastolic function in patients with essential hypertension. Am J Hypertens.

[14] Dormandy JA, Charbonnel B, Eckland DJ, Erdmann E, Massi-Benedetti M, Moules IK, Skene AM, Tan MH, Lefebvre PJ, Murray GD (2005). Secondary prevention of macrovascular events in patients with type 2 diabetes in the PROactive Study (PROspective pioglitAzone Clinical Trial In macroVascular Events): a randomised controlled trial. Lancet..

[15] Scirica BM, Bhatt DL, Braunwald E, Steg PG, Davidson J, Hirshberg B, Ohman P, Frederich R, Wiviott SD, Hoffman EB (2013). Saxagliptin and cardiovascular outcomes in patients with type 2 diabetes mellitus. N Engl J Med.

[16] Zinman B, Wanner C, Lachin JM, Fitchett D, Bluhmki E, Hantel S, Mattheus M, Devins T, Johansen OE, Woerle HJ (2015). Empagliflozin, cardiovascular outcomes, and mortality in type 2 diabetes. N Engl J Med.

[17] Gautam S, Agiro A, Barron J, Power T, Weisman H, White J (2017). Heart failure hospitalization risk associated with use of two classes of oral antidiabetic medications: an observational, real-world analysis. Cardiovasc Diabetol.

[18] Neal B, Perkovic V, Mahaffey KW, de Zeeuw D, Fulcher G, Erondu N, Shaw W, Law G, Desai M, Matthews DR (2017). Canagliflozin and cardiovascular and renal events in type 2 diabetes. N Engl J Med..

[19] Mahaffey KW, Neal B, Perkovic V, de Zeeuw D, Fulcher G, Erondu N, Shaw W, Fabbrini E, Sun T, Li Q (2017). Canagliflozin for primary and secondary prevention of cardiovascular events: results from the CANVAS Program (Canagliflozin Cardiovascular Assessment Study). Circulation..

[20] Lang RM, Bierig M, Devereux RB, Flachskampf FA, Foster E, Pellikka PA, Picard MH, Roman MJ, Seward J, Shanewise JS (2005). Recommendations for chamber quantification: a report from the American Society of Echocardiography’s Guidelines and Standards Committee and the Chamber Quantification Writing Group, developed in conjunction with the European Association of Echocardiography, a branch of the European Society of Cardiology. J Am Soc Echocardiogr.

[21] Ommen SR, Nishimura RA, Appleton CP, Miller FA, Oh JK, Redfield MM, Tajik AJ (2000). Clinical utility of Doppler echocardiography and tissue Doppler imaging in the estimation of left ventricular filling pressures: a comparative simultaneous Doppler-catheterization study. Circulation.

[22] La Rovere MT, Pinna GD, Raczak G (2008). Baroreflex sensitivity: measurement and clinical implications. Ann Noninvasive Electrocardiol..

[23] Malliani A, Pagani M, Lombardi F (1994). Physiology and clinical implications of variability of cardiovascular parameters with focus on heart rate and blood pressure. Am J Cardiol.

[24] Ferrannini E, Baldi S, Frascerra S, Astiarraga B, Barsotti E, Clerico A, Muscelli E (2017). Renal handling of ketones in response to sodium-glucose cotransporter 2 inhibition in patients with type 2 diabetes. Diabetes Care.

[25] Sano M, Takei M, Shiraishi Y, Suzuki Y (2016). Increased hematocrit during sodium-glucose cotransporter 2 inhibitor therapy indicates recovery of tubulointerstitial function in diabetic kidneys. J Clin Med Res.

[26] Heerspink HJL, de Zeeuw D, Wie L, Leslie B, List J (2013). Dapagliflozin a glucose-regulating drug with diuretic properties in subjects with type 2 diabetes. Diabetes Obes Metab.

[27] O’Neill J, Fasching A, Pihl L, Patinha D, Franzen S, Palm F (2015). Acute SGLT inhibition normalizes O2 tension in the renal cortex but causes hypoxia in the renal medulla in anaesthetized control and diabetic rats. Am J Physiol Renal Physiol.

[28] Hirata A, Minamino T, Asanuma H, Fujita M, Wakeno M, Myoishi M, Tsukamoto O, Okada K, Koyama H, Komamura K (2006). Erythropoietin enhances neovascularization of ischemic myocardium and improves left ventricular dysfunction after myocardial infarction in dogs. J Am Coll Cardiol.

[29] van der Meer P, Lipsic E (2006). Erythropoietin: repair of the failing heart. J Am Coll Cardiol.

[30] Namiuchi S, Kagaya Y, Ohta J, Shiba N, Sugi M, Oikawa M, Kunii H, Yamao H, Komatsu N, Yui M (2005). High serum erythropoietin level is associated with smaller infarct size in patients with acute myocardial infarction who undergo successful primary percutaneous coronary intervention. J Am Coll Cardiol.

[31] Parsa CJ, Matsumoto A, Kim J, Riel RU, Pascal LS, Walton GB, Thompson RB, Petrofski JA, Annex BH, Stamler JS (2003). A novel protective effect of erythropoietin in the infarcted heart. J Clin Investig.

[32] Parissis JT, Kourea K, Panou F, Farmakis D, Paraskevaidis I, Ikonomidis I, Filippatos G, Kremastinos DT (2008). Effects of darbepoetin alpha on right and left ventricular systolic and diastolic function in anemic patients with chronic heart failure secondary to ischemic or idiopathic dilated cardiomyopathy. Am Heart J..

[33] Pappas KD, Gouva CD, Katopodis KP, Nikolopoulos PM, Korantzopoulos PG, Michalis LK, Goudevenos JA, Siamopoulos KC (2008). Correction of anemia with erythropoietin in chronic kidney disease (stage 3 or 4): effects on cardiac performance. Cardiovasc Drugs Ther.

[34] Martini J, Tsai AG, Cabrales P, Johnson PC, Intaglietta M (2006). Increased cardiac output and microvascular blood flow during mild hemoconcentration in hamster window model. Am J Physiol Heart Circ Physiol.

[35] Ferrannini E, Mark M, Mayoux E (2016). CV protection in the EMPA-REG OUTCOME trial: a “Thrifty Substrate” hypothesis. Diabetes Care.

[36] Heise T, Jordan J, Wanner C, Heer M, Macha S, Mattheus M, Lund SS, Woerle HJ, Broedl UC (2016). Pharmacodynamic effects of single and multiple doses of empagliflozin in patients with type 2 diabetes. Clin Ther.

[37] Oh J, Kang SM, Hong N, Youn JC, Han S, Jeon ES, Cho MC, Kim JJ, Yoo BS, Chae SC (2013). Hemoconcentration is a good prognostic predictor for clinical outcomes in acute heart failure: data from the Korean Heart Failure (KorHF) Registry. Int J Cardiol.

[38] Greene SJ, Gheorghiade M, Vaduganathan M, Ambrosy AP, Mentz RJ, Subacius H, Maggioni AP, Nodari S, Konstam MA, Butler J (2013). Haemoconcentration, renal function, and post-discharge outcomes among patients hospitalized for heart failure with reduced ejection fraction: insights from the EVEREST trial. Eur J Heart Fail.

[39] Pham SV, Chilton RJ (2017). EMPA-REG OUTCOME: the cardiologist’s point of view. Am J Cardiol.

[40] Rajasekeran H, Lytvyn Y, Cherney DZ (2016). Sodium-glucose cotransporter 2 inhibition and cardiovascular risk reduction in patients with type 2 diabetes: the emerging role of natriuresis. Kidney Int.

[41] Inzucchi SE, Zinman B, Wanner C, Ferrari R, Fitchett D, Hantel S, Espadero R-M, Woerle H-J, Broedl UC, Johansen OE (2015). SGLT-2 inhibitors and cardiovascular risk: proposed pathways and review of ongoing outcome trials. Diabetes Vasc Dis Res.

[42] Sato T, Aizawa Y, Yuasa S, Kishi S, Fuse K, Fujita S, Ikeda Y, Kitazawa H, Takahashi M, Sato M (2018). The effect of dapagliflozin treatment on epicardial adipose tissue volume. Cardiovasc Diabetol.

[43] Bouchi R, Terashima M, Sasahara Y, Asakawa M, Fukuda T, Takeuchi T, Nakano Y, Murakami M, Minami I, Izumiyama H (2017). Luseogliflozin reduces epicardial fat accumulation in patients with type 2 diabetes: a pilot study. Cardiovasc Diabetol.

[44] Solini A, Giannini L, Seghieri M, Vitolo E, Taddei S, Ghiadoni L, Bruno RM (2017). Dapagliflozin acutely improves endothelial dysfunction, reduces aortic stiffness and renal resistive index in type 2 diabetic patients: a pilot study. Cardiovasc Diabetol.

[45] Shigiyama F, Kumashiro N, Miyagi M, Ikehara K, Kanda E, Uchino H, Hirose T (2017). Effectiveness of dapagliflozin on vascular endothelial function and glycemic control in patients with early-stage type 2 diabetes mellitus: DEFENCE study. Cardiovasc Diabetol.

[46] Kusaka H, Koibuchi N, Hasegawa Y, Ogawa H, Kim-Mitsuyama S (2016). Empagliflozin lessened cardiac injury and reduced visceral adipocyte hypertrophy in prediabetic rats with metabolic syndrome. Cardiovasc Diabetol.

[47] Wenzel RR, Bruck H, Noll G, Schafers RF, Daul AE, Philipp T (2000). Antihypertensive drugs and the sympathetic nervous system. J Cardiovasc Pharmacol.

[48] Yoshikawa T, Kishi T, Shinohara K, Takesue K, Shibata R, Sonoda N, Inoguchi T, Sunagawa K, Tsutsui H, Hirooka Y (2017). Arterial pressure lability is improved by sodium-glucose cotransporter 2 inhibitor in streptozotocin-induced diabetic rats. Hypertens Res..

[49] Tomai F, Crea F, Gaspardone A, Versaci F, De Paulis R, de Penta Peppo A, Chiariello L, Gioffre PA (1994). Ischemic preconditioning during coronary angioplasty is prevented by glibenclamide, a selective ATP-sensitive K+ channel blocker. Circulation.

[50] Mita T, Katakami N, Shiraiwa T, Yoshii H, Onuma T, Kuribayashi N, Osonoi T, Kaneto H, Kosugi K, Umayahara Y (2016). Sitagliptin attenuates the progression of carotid intima-media thickening in insulin-treated patients with type 2 diabetes: The Sitagliptin Preventive Study of Intima-Media Thickness Evaluation (SPIKE): a randomized controlled trial. Diabetes Care.

[51] Ida S, Murata K, Betou K, Kobayashi C, Ishihara Y, Imataka K, Uchida A, Monguchi K, Kaneko R, Fujiwara R (2016). Effect of trelagliptin on vascular endothelial functions and serum adiponectin level in patients with type 2 diabetes: a preliminary single-arm prospective pilot study. Cardiovasc Diabetol.

[52] Verma S, Garg A, Yan AT, Gupta AK, Al-Omran M, Sabongui A, Teoh H, Mazer CD, Connelly KA (2016). Effect of empagliflozin on left ventricular mass and diastolic function in individuals with diabetes: an important clue to the EMPA-REG OUTCOME trial?. Diabetes Care.

[53] Patorno E, Goldfine AB, Schneeweiss S, Everett BM, Glynn RJ, Liu J, Kim SC (2018). Cardiovascular outcomes associated with canagliflozin versus other non-gliflozin antidiabetic drugs: population based cohort study. BMJ.

[54] Inzucchi SE, Zinman B, Fitchett D, Wanner C, Ferrannini E, Schumacher M, Schmoor C, Ohneberg K, Johansen OE, George JT (2017). How does empagliflozin reduce cardiovascular mortality? Insights from a mediation analysis of the EMPA-REG OUTCOME trial. Diabetes care.

[55] Benjamin EJ, Blaha MJ, Chiuve SE, Cushman M, Das SR, Deo R, de Ferranti SD, Floyd J, Fornage M (2017). Heart disease and stroke statistics—2017 update: a report from the American Heart Association. Circulation..

[56] Kane GC, Karon BL, Mahoney DW, Redfield MM, Roger VL, Burnett JC, Jacobsen SJ, Rodeheffer RJ (2011). Progression of left ventricular diastolic dysfunction and risk of heart failure. JAMA.

[57] Lund LH, Benson L, Dahlstrom U, Edner M, Friberg L (2014). Association between use of beta-blockers and outcomes in patients with heart failure and preserved ejection fraction. JAMA.

[58] Massie BM, Carson PE, McMurray JJ, Komajda M, McKelvie R, Zile MR, Anderson S, Donovan M, Iverson E, Staiger C (2008). Irbesartan in patients with heart failure and preserved ejection fraction. N Engl J Med.

[59] Solomon SD, Claggett B, Lewis EF, Desai A, Anand I, Sweitzer NK, O’Meara E, Shah SJ, McKinlay S, Fleg JL (2016). Influence of ejection fraction on outcomes and efficacy of spironolactone in patients with heart failure with preserved ejection fraction. Eur Heart J.

[60] Holland DJ, Kumbhani DJ, Ahmed SH, Marwick TH (2011). Effects of treatment on exercise tolerance, cardiac function, and mortality in heart failure with preserved ejection fraction. A meta-analysis. J Am Coll Cardiol.

[61] CONSENSUS Trial Study Group (1987). Effects of enalapril on mortality in severe congestive heart failure. Results of the Cooperative North Scandinavian Enalapril Survival Study (CONSENSUS). N Engl J Med.

