# Supplementary material for: Effect of canagliflozin on left ventricular diastolic function in patients with type 2 diabetes
Source: Cardiovasc Diabetol. 2018 May 22;17:73. doi: 10.1186/s12933-018-0717-9 (PMC5963148; doi:10.1186/s12933-018-0717-9)
Supplement: Supplementary file 1 — Additional file 1: Table S1. Comparison of echocardiographic findings between baseline and at the end of the 3-months study period in the total population, the primary, and secondary prevention. [file 12933_2018_717_MOESM1_ESM.docx]

**Comparison of echocardiographic findings between baseline and 3 months after start of the study treatment in the total population, the primary, and secondary prevention**

| **Additional file 1: Table S1** Comparison of echocardiographic findings between baseline and at the end of the 3- months study period in the total population, the primary, and secondary prevention | | | | | | | | | | | | |
| --- | --- | --- | --- | --- | --- | --- | --- | --- | --- | --- | --- | --- |
|  | No. (%) | Pre Canagliflozin | | | | Post Canagliflozin | | | | *p* value (Pre vs. Post) | | *p* value (Primary vs. Secondary) |
| Septal E/e’ |  |  |  |  |  | |  |  |  | |  | |
| Primary prevention | 12 (34) | 13.0 | ± | 3.3 | 11.8 | | ± | 2.4 | 0.014 | | 0.277 | |
| Secondary prevention | 25 (64) | 15.2 | ± | 3.7 | 12.7 | | ± | 3.6 | 0.039 | |  |  |
| Overall population |  | 13.7 | ± | 3.5 | 12.1 | | ± | 2.8 | 0.001 | |  | |
| LV mass index (g/m^2^) |  |  |  |  |  | |  |  |  | |  | |
| Primary prevention |  | 82.3 | ± | 16.1 | 78.3 | | ± | 15.6 | 0.041 | | 0.413 | |
| Secondary prevention |  | 81.3 | ± | 15.8 | 75.4 | | ± | 18.7 | 0.037 | |  |  |
| Overall population |  | 82.0 | ± | 15.8 | 77.3 | | ± | 16.4 | 0.003 | |  | |
| EF (%) |  |  |  |  |  | |  |  |  | |  | |
| Primary prevention |  | 66.2 | ± | 5.3 | 65.1 | | ± | 5.6 | 0.308 | | 0.233 | |
| Secondary prevention |  | 64.7 | ± | 4.4 | 65.8 | | ± | 5.4 | 0.432 | |  |  |
| Overall population |  | 65.7 | ± | 5.0 | 65.3 | | ± | 5.5 | 0.652 | |  | |
| Data are mean ± SD. The paired *t* test was used to comparison of echocardiographic findings between baseline and at 3 months. The Student’s *t*-test was used to compare mean Δseptal E/e` between primary prevention group and secondary prevention group. SD, standard deviation; E, velocity of early mitral flow; e′, early peak velocity of annulus; LV, left ventricular; EF, ejection fraction | | | | | | | | | | | | |

Compared to baseline levels, septal E/e` and LV mass index significantly decreased in the overall population group, primary prevention group, and secondary prevention group. Furthermore, there was no difference in treatment effect between the primary and secondary prevention groups. However, EF did not improve in these groups.
